# Identification of tumor associated neutrophils-related genes in triple-negative breast cancer for predicting prognosis and therapeutic response through integrated single-cell analysis

**DOI:** 10.3389/fimmu.2025.1613529

**Published:** 2025-09-22

**Authors:** Koukou Li, Lingli Gong, Yuxuan Zhang, Lihua Yang, Daxing Xu, Mei Wang, Yaling Hu

**Affiliations:** ^1^ Department of Laboratory Medicine, The Affiliated Wuxi People’s Hospital of Nanjing Medical University, Wuxi People’s Hospital, Wuxi Medical Center, Nanjing Medical University, Wuxi, Jiangsu, China; ^2^ Center of Clinical Research, The Affiliated Wuxi People’s Hospital of Nanjing Medical University, Wuxi People’s Hospital, Wuxi Medical Center, Nanjing Medical University, Wuxi, China; ^3^ Department of Pathology, The Affiliated Wuxi People’s Hospital of Nanjing Medical University, Wuxi, China

**Keywords:** triple-negative breast cancer, immuno-regulation, metastasis, single-cell transcriptional analysis, tumor associated neutrophils

## Abstract

Tumor-associated neutrophils (TANs) significantly influence tumor development, immune system suppression, and the spread of cancer in triple-negative breast cancer (TNBC). However, their molecular pathways and potential for therapy are not completely understood. We utilized Seurat and Harmony to perform quality control, batch correction, and cell annotation on single-cell RNA-seq data from TNBC patients (GSE222854). Comprehensive bioinformatics approaches—including immune infiltration analysis, GSEA, GSVA, drug sensitivity profiling, and ligand-receptor interaction network analysis were combined with functional validation (colony formation and Transwell assays) and clinical correlation studies via polychromatic immunofluorescence. Four TAN-associated genes (RASGRP4, TIMM10B, TNFRSF13C, and GRAP) with distinct roles in TNBC progression were identified. Functional assays revealed pro-tumorigenic effects of RASGRP4, TIMM10B, and GRAP, whereas TNFRSF13C exhibited tumor-suppressive properties. Clinically, elevated RASGRP4 and TIMM10B expression with reduced TNFRSF13C expression correlated with poor survival and accelerated disease progression, underscoring their prognostic significance. Our study revealed RASGRP4, TIMM10B, and TNFRSF13C as promising therapeutic targets in TNBC. Targeting these TAN-associated genes may disrupt pro-tumor immune responses, suggesting novel strategies to improve patient outcomes.

## Introduction

Triple-negative breast cancer (TNBC) is characterized by the loss of estrogen receptor (ER), progesterone receptor (PR) and epidermal growth factor receptor 2 (HER2) expression. This represents approximately 10-20% of all cases of breast cancer (1), and is associated with poor differentiation and a high recurrence rate (2, 3). Compared with HER2-positive or hormone receptor-positive breast cancers, TNBC shows a limited response to targeted therapies and immunotherapies, with a median survival of only 13 months (4–6). Therefore, innovative approaches are urgently needed to decipher the basic mechanisms underlying TNBC progression and to create more effective treatment strategies.

Within the tumor microenvironment (TME), infiltrating immune cells such as B cells, T cells, NK cells, macrophages, neutrophils, and dendritic cells are recognized as critical determinants of progression and prognosis in breast cancer patients (7–9). Among these immune components, tumor-associated neutrophils (TANs) are notably prevalent and are postulated to possess anti-tumor (N1) and pro-tumor (N2) functions, thereby affecting patient outcomes (9–11). They contribute to TNBC cell proliferation by increasing a proliferation-inducing ligand (APRIL) and releasing neutrophil elastase (NE) (12, 13). In addition, TANs facilitate tumor angiogenesis through the release of vascular endothelial growth factor (VEGF), chemokines, and matrix metalloproteinase-9 (MMP-9) (14). They also contact circulating tumor cells (CTCs), resulting in the formation of neutrophil extracellular traps (NETs), which facilitate blood-borne metastasis, particularly to the lungs (15–18). While inhibiting neutrophil infiltration can suppress the growth and metastasis of TNBC, neutrophils are the most abundant immune cells in the body, and blocking them entirely may impair immune defense. Thus, the selective targeting and elimination of N2 TANs is emerging as a promising therapeutic strategy for TNBC, aiming to decrease tumor progression without impairing overall immunity.

Understanding the heterogeneity and identifying the biomarkers of TANs holds substantial potential for improving survival prediction and guiding TAN-based therapeutic strategies for TNBC. Traditional bulk RNA sequencing (RNA-seq) methods are inadequate for accurately profiling the transcriptional characteristics of specific cell types (19, 20). However, new advances in single-cell RNA sequencing (scRNA-seq) have shed novel light on cell diversity at the single-cell level and facilitated the classification of distinct cell types within an organ (15, 16). This technological innovation presents a unique opportunity to bridge the knowledge gap by enabling detailed dissection of cellular modeling. Numerous scRNA-seq studies have been conducted on breast cancer, with some specifically targeting individual cell populations in TNBC, such as B cells (21), exhausted T cells, M2 macrophages (22, 23), and fibroblasts (24). Other studies have characterized the general features of immune cells (25) or provided a comprehensive depiction of the tumor microenvironment by sequencing the total cell population isolated from breast tumors (26, 27). Nevertheless, scRNA-seq investigations explicitly aimed at elucidating the immunosuppressive roles of TANs in TNBC have yet to be reported.

In this study, we integrated published single-cell datasets from TNBC and identified neutrophil subsets and their marker genes. Combining functional analysis with clinical validation, we pinpointed translocase of inner mitochondrial membrane 10B (TIMM10B), Ras guanyl nucleotide-releasing protein 4 (RASGRP4), and transmembrane activator and calcium modulator and cyclophilin ligand interactor (TNFRSF13C) as potential genes involved in TAN-mediated tumorigenesis in TNBC.

## Materials and methods

### Data resources

The GEO database (28) supplied the single-cell dataset GSE222854, which contains peripheral blood single-cell expression profiles from two triple-negative breast cancer cases for examination. This dataset specifically focuses on CD45^+^ Ter119^-^ cells, thereby excluding erythroid lineage cells. This selection strategy facilitates targeted investigation of immune components, particularly tumor-associated neutrophils, within the tumor microenvironment. Additionally, bulk transcriptomic data from 115 triple-negative breast cancer samples were obtained from the TCGA database for complementary analysis.

### Quality control

Using the Seurat package, the scRNA-seq profile was initially analyzed, with cells filtered according to several criteria, such as the total unique molecular identifier (UMI) for each cell, the quantity of expressed genes and the percentage of mitochondrial and ribosomal gene expression per cell. Theproportion of mitochondrial and ribosomal gene expression was defined as the percentage of the total expression attributed to these genes relative to the overall gene expression. Cells exhibiting elevated levels of mitochondrial and ribosomal gene expression tend to have low RNA expression, indicating that these cells are undergoing apoptotic processes. For quality control, we employed the median absolute deviation (MAD) method. Conventionally, any variable exceeding 3-MADs from the median was classified as an outlier and was excluded from analysis.

### Dimension reduction, clustering and cell annotation

During analysis, dimension reduction, clustering, and cell annotation were performed using established methodologies. The ‘LogNormalize’ method, recognized worldwide, was applied by scaling the total expression in each cell to 10,000 using a coefficient S0, followed by a logarithmic transformation for standardization. Cell cycle scores were computed utilizing the CellCycleScoring function. To identify highly variable genes, the Find Variable Features function was applied. Furthermore, the ScaleData function was utilized to mitigate gene expression variability attributed to differing proportions of mitochondrial gene expression, ribosomal gene expression, and cell cycle effects. The expression matrix was subsequently subjected to linear reduction via RunPCA, with principal components subsequently selected for further analysis. To mitigate batch effects, Harmony was applied, and nonlinear dimensionality reduction was achieved through the RunUMAP Unified Manifold Approximation and Projection (UMAP) approach. For cell annotation, cell types and their marker genes in the tissues were identified mainly through the CellMarker and PanglaoDB databases and relevant literature, supplemented by automated annotation using the SingleR software.

### Random survival forest method analysis

Using the randomForestSRC software package, we selected features and evaluated the significance of prognosis-related genes with the random survival forest algorithm, performing 1000 iterations in a Monte Carlo simulation. If the relative importance of genes was above 0.2, they were categorized as final marker genes.

### Immune cell infiltration analysis

To evaluate the types of immune cells in the microenvironment, the CIBERSORT approach, which applies support vector regression to deconvolute the expression matrix of immune cell subtypes, is commonly utilized. This method includes 547 biomarkers that distinguish 22 types of human immune cells, such as T cells, B cells, plasma cells, and different myeloid cell subsets. The CIBERSORT algorithm was used to analyze patient data and ascertain the relative proportions of 22 infiltrating immune cell types. A correlation analysis was performed to study the connections.

### Drug sensitivity analysis

By accessing the Genomics of Drug Sensitivity in Cancer database (GDSC), the most comprehensive repository of cancer drug sensitivity information, available at https://www.cancerrxgene.org/, we used the ‘oncoPredict’ package in R to analyze the sensitivity of individual tumor samples to chemotherapy. The half-maximal inhibitory concentration (IC50) values for each chemotherapeutic agent were derived through regression analysis. The accuracy of the regression and prediction models was evaluated through tenfold cross-validation on the GDSC training dataset. In the analysis, default configurations were implemented, which included using ‘combat’ to address batch effects and averaging repeated gene expression readings.

### Gene set enrichment analysis

The classification of patients into high and low expression categories was performed according to the levels of important genes. Subsequent analysis of the differences in signaling pathways between these two groups was conducted using GSEA. The gene sets used as a background were annotated from version 7.0 of the Molecular Signatures Database (MSigDB) and served as annotation gene sets for subtype pathways. Pathways were subjected to differential expression analysis by group, and gene sets with significant enrichment (adjusted p-value under 0.05) were ranked by consistency scores. GSEA is frequently employed to investigate intricate associations within biological data.

### Gene set variation analysis

GSVA is a nonparametric, unsupervised approach utilized for assessing the enrichment of gene sets within a transcriptome. By systematically scoring the gene set of interest, GSVA transforms gene-level variations into pathway-level alterations, thereby facilitating the evaluation of the biological functions of the sample. In this research, gene sets from the Molecular Signatures Database were utilized along with the GSVA algorithm to produce detailed scores for each gene set, allowing for the evaluation of possible biological functional variations across different samples.

### Nomogram and correction curve of key genes participating in the construction

By employing regression analysis, a nomogram was created on a single plane with scaled linear segments that illustrate gene expression and clinical symptoms, highlighting the relationships between variables in the predictive model. Through the use of a multifactor regression model, scores were given to each level of influencing factors based on their contribution to the outcome variable, as determined by the magnitude of the regression coefficient. The total score was subsequently calculated to determine the predicted value.

### Ligand receptor interaction analysis

CellCall is an all-encompassing toolkit aimed at deducing intercellular communication networks and internal regulatory signals by merging intracellular and intercellular signals. It assembles datasets of ligand–receptor–transcription factor (L-R-TF) axes from KEGG pathway results. By utilizing established L-R-TF interaction knowledge, CellCall infers intercellular communication through the integration of ligand and receptor expression data with the downstream activity of transcription factors linked to specific L-R pairs.

### Quasitemporal analysis

Studies conducted at the single-cell level have facilitated the characterization of transcriptional regulation within complex physiological processes and highly heterogeneous cell populations. These investigations have been instrumental in identifying genes that are specific to particular cell subtypes, genes that serve as markers for intermediate stages of biological processes, and genes that mediate transitions between distinct cell fates. In numerous studies focusing on single cells, gene expression is not synchronized, with each cell serving as a distinct temporal snapshot of the transcription process being examined. The Monocle algorithm introduced a strategic approach to address this complexity.

### Cell culture

MDA-MB-231 cells were obtained from Immocell Biotechnology (IM-H026, Xiamen, China). HL-60 cells were obtained from Anweisci (AW-CH0142, Shanghai, China). MDA-MB-231 cells were grown in DMEM (PYG0073, Boster Biotech, Wuhan, China) supplemented with 10% FBS (40-001-1ACS, BI, Israel) and 1% penicillin–streptomycin (BL505A, Biosharp, Hefei, China). HL-60 cells were cultured in RPIM 1640 (PYG0122, Boster Biotech, Wuhan, China) supplemented with 20% FBS and 1% penicillin–streptomycin. To induce neutrophil-like differentiation in HL-60 cells, 1% DMSO (1084ML500, BioFroxx, Germany) was added to the culture media for a period of 6 days. For coculture experiments, we employed 6-well transwell plates (703001, NEST, China) featuring polyester membrane inserts (0.4 µm pores, TCS001006, Jet, China), with dHL-60 cells in the upper compartments and MDA-MB-231 cells in the lower compartment.

### Transient overexpression and knockdown of genes in dHL-60 cells

Full-length plasmids for overexpressing RASGRP4, TIMM10B, TNFRSF13C, and GRAP were purchased from MiaoLing Plasmids (Wuhan, China). An empty vector served as a negative control. The plasmids were temporarily introduced into dHL60 cells using Lipofectamine 3000 (L300015, Thermo Fisher, USA) following the manufacturer’s instructions.

### RNA extraction and quantitative real-time polymerase chain reaction

TRIzol reagent (Invitrogen) was used to extract total RNA, and reverse transcription was carried out using the M-MLV Reverse Transcriptase Kit from CWBIO, Beijing, China. Gene transcript levels were measured relative to that of control gene (GAPDH) using Real SYBR Mixture (Q711-02, Vazyme, China). The qRT-PCR primers used are listed in **Table 1**.

**Table 1:** List of primers used for qRT-PCR.

Gene name	Forward primer (5’-3’)	Reverse primer (5’-3’)
GAPDH	CTGGGCTACACTGAGCACC	AAGTGGTCGTTGAGGGCAATG
TIMM10B	GAGACGGGGTTTCACTGTAT	TCCCATCCTCCTATCTTCTG
GRAP	CTCCATCTCTGTCAGGCATGA	TGTCCTGTAGTAGTCTACCAGC
TNFRSF13C	GAACTCCTGACCTTGTGATC	TTCCCATCCTCCTATCTTCTG
RASGRP4	GCACCGGAAAAATAGGAGGG	CAGCACCATGTTGAGCATGT

### Colony formation assay

A cell suspension of two milliliters, containing 1 × 10^3^ MDA-MB-231 cells, was placed in each well of 6-well plates and cultured for 10 days with supernatants from control and RASGRP4/TIMM10B/TNFRSF13C/GRAP overexpressing dHL-60 cells. The colonies were fixed with 4% PFA (BL539A, Biosharp, Hefei, China) for 15 minutes, followed by staining with 0.5% crystal violet (E607309-0100, Sangon Biotech Co., Ltd, Shanghai, China) for another 15 minutes. Colonies with more than 50 cells were counted.

### Transwell migration assay

After co-culturing MDA-MB-231 cells with control or RASGRP4-, TIMM10B-, TNFRSF13C-, or GRAP-overexpressing dHL-60 cells at a ratio of 1:10 (dHL-60: MDA-MB-231) for three days, the MDA-MB-231 cells were harvested, resuspended in serum-free medium, and seeded into the upper chambers of Transwell inserts (8 μm pore size; 3422, Corning, USA) at a density of 1 × 10^4^ cells in 100 μL per well. The lower chambers were filled with 600 μL of complete medium containing 10% FBS as a chemoattractant. Cells were allowed to migrate at 37 °C for 24 hours. After incubation, non-migrated cells on the upper side of the membrane were gently removed, and the migrated cells on the lower side were fixed with 4% paraformaldehyde and stained with 0.5% crystal violet. Migrated cells were counted under a light microscope in five randomly selected fields.

### Polychromatic immunofluorescence staining

Tissue microarray slides were purchased from Superchip Outdo Company, and the study was approved by the Ethics Committee of Shanghai Outdo Biotech Company (HBreD120CS01). Next the tissue samples were subjected to polychromatic immunofluorescence staining using a six-color multiplex fluorescence immunohistochemical staining kit (Absin, abs50014). To prevent nonspecific binding, the slides were first blocked using Tris-buffered saline with Tween 20 and 5% serum before antibody incubation. The primary antibodies used in the experiment included antibodies against MPO (ZSGB-Bio, ZA-0197, diluted1:200), TIMM10B (Proteintech, 10907-1, diluted 1:200), GRAP (Proteintech, 14505-1, diluted 1:200), and TNFRSF13C (Proteintech, 22582-1, diluted 1:200), RASGRP4 (Abcam, 96293, diluted 1:100). Following incubation with the appropriate antibodies, nuclei were counterstained with DAPI, and the slides were sealed and scanned using a fluorescence scanner to capture high-resolution images. The expression levels of MPO, TIMM10B, GRAP, TNFRSF13C, and RASGRP4 were evaluated through semiquantitative analysis, and IHC scores were assigned based on both the staining intensity and the percentage of positively stained cells.

### Statistical analysis

Statistical analyses were performed using R (version 4.3.0), with the significance level set at *P* < 0.05. The data are shown as the mean ± standard deviation (SD) from at least three separate experiments. Statistical significance was assessed using a one-way ANOVA, with *P < 0.05, **P < 0.01, ***P < 0.001, and ****P < 0.0001 regarded as statistically significant in compared with the control group.

## Results

### Single cell transcriptome profiling of TNBC

To obtain high quality data across different samples, cells with results capturing fewer than 50 genes were not included in the analysis. The following criteria were used for filtering: the mitochondrial content (mt) was required to be less than or equal to the median plus three times the median absolute deviation (3MAD); the number of detected genes (nFeature_RNA) was required to be less than or equal to the median plus 3MAD; and the total number of unique molecular identifiers (nCount_RNA) was required to be less than or equal to the median plus 3MAD. Additionally, cells whose nFeature_RNA was greater than 50 and whose mitochondrial content (percent.mt) was less than or equal to the median plus 3MAD were retained. Here, nFeature_RNA is the measure of genes detected, nCount_RNA represents the total number of unique molecular identifiers per cell, percent.mt indicates the fraction of mitochondrial reads, and percent.ribo represents the fraction of ribosomal reads. The filtered data are presented in both a violin plot and a scatter plot (**Supplementary Figure S1A**). In all, we discovered 2,000 highly variable genes (**Supplementary Figure S1B**) were discovered after completing data normalization, homogenization, PCA, and harmony processing (**Supplementary Figures S1C–F**).

**Figure 1:**
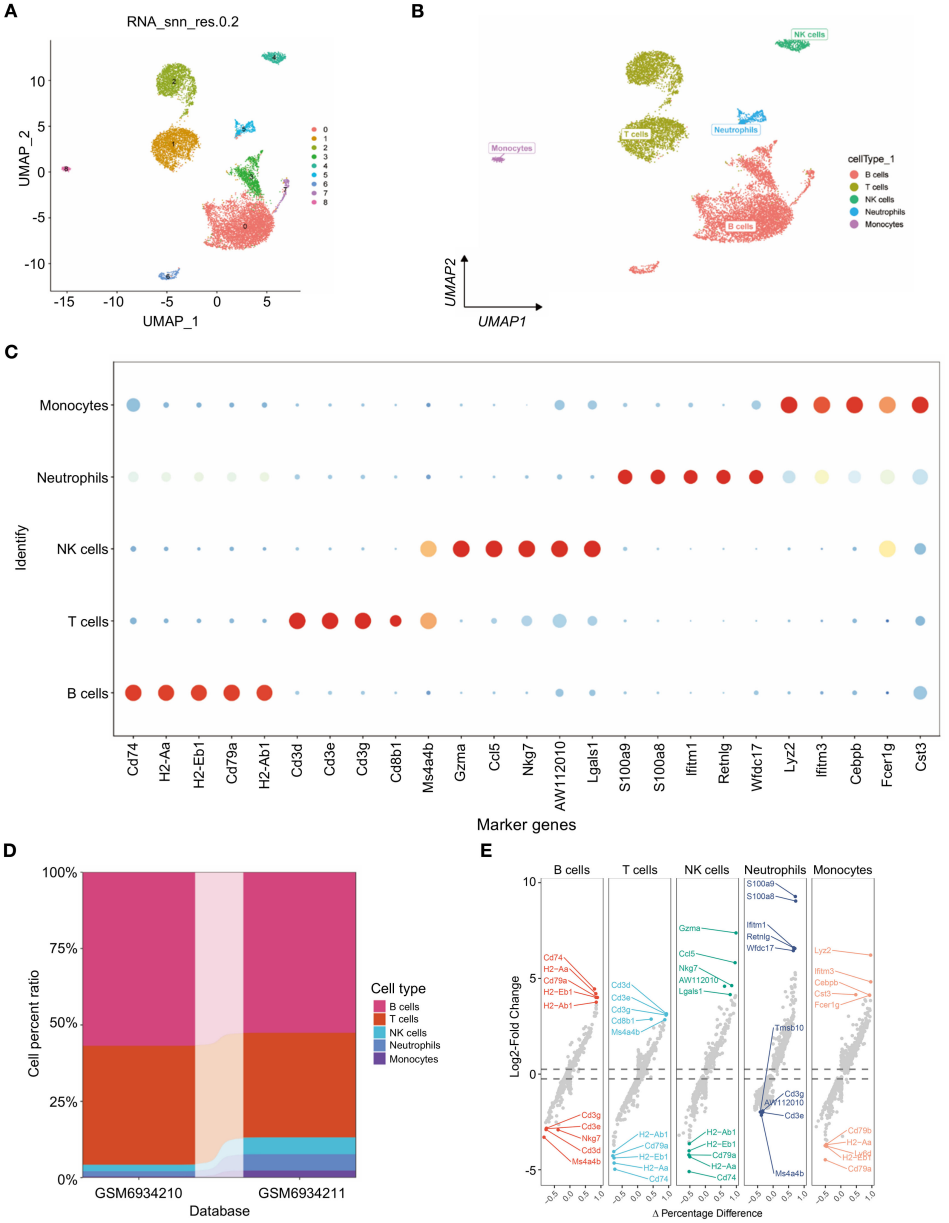
Single-cell transcriptome profiling of triple-negative breast cancer. **(A)** scRNA-seq data from 12,316 triple-negative breast cancer cells were subjected to UMAP. **(B)** UMAP visualization of the scRNAseq data showing all the cells, with clusters indicated by colors and labeled based on inferred cell types. **(C)** Dot plot showing the expression of known marker genes in these five clusters. Dot size reflects each gene’s expressing percentage of each cluster’s cells; Dot color represents the expression level. **(D)** Proportions of B cells, T cells, NK cells, neutrophils, and monocytes. **(E)** For each cell type, volcano plots display the fold change in gene expression (log2FC) against the percent difference in expression, highlighting genes with significant differential expression.

Furthermore, nine distinct cell subtypes were identified through uniform manifold approximation and projection (UMAP) analysis (**Figure 1A**). Each subtype underwent annotation, resulting in the classification of all clusters into five major cell categories: B cells, T cells, NK cells, neutrophils, and monocytes (as illustrated in **Figures 1A, B**). A bubble diagram depicting the classical markers for these five cell types is presented in **Figure 1C**, while **Figure 1D** provides a bar diagram illustrating the cell proportions across various subsamples. The differential expression of the marker genes of neutrophils, i.e., S100a9, S100a8, Ifitm1, Retnlg and Wfdc17, was visualized (**Figure 1C**). Additionally, we conducted an analysis of cell subtype differences at the single-cell level utilizing the markerVocalno function from the scRNAtoolVis package. This approach identified cluster-specific marker genes for each cell type, aiding in precise cell annotation and deepening our understanding of tumor microenvironment heterogeneity in triple-negative breast cancer (**Figure 1E**).

### Identification of candidate genes through random survival forest analysis

**Figure 2:**
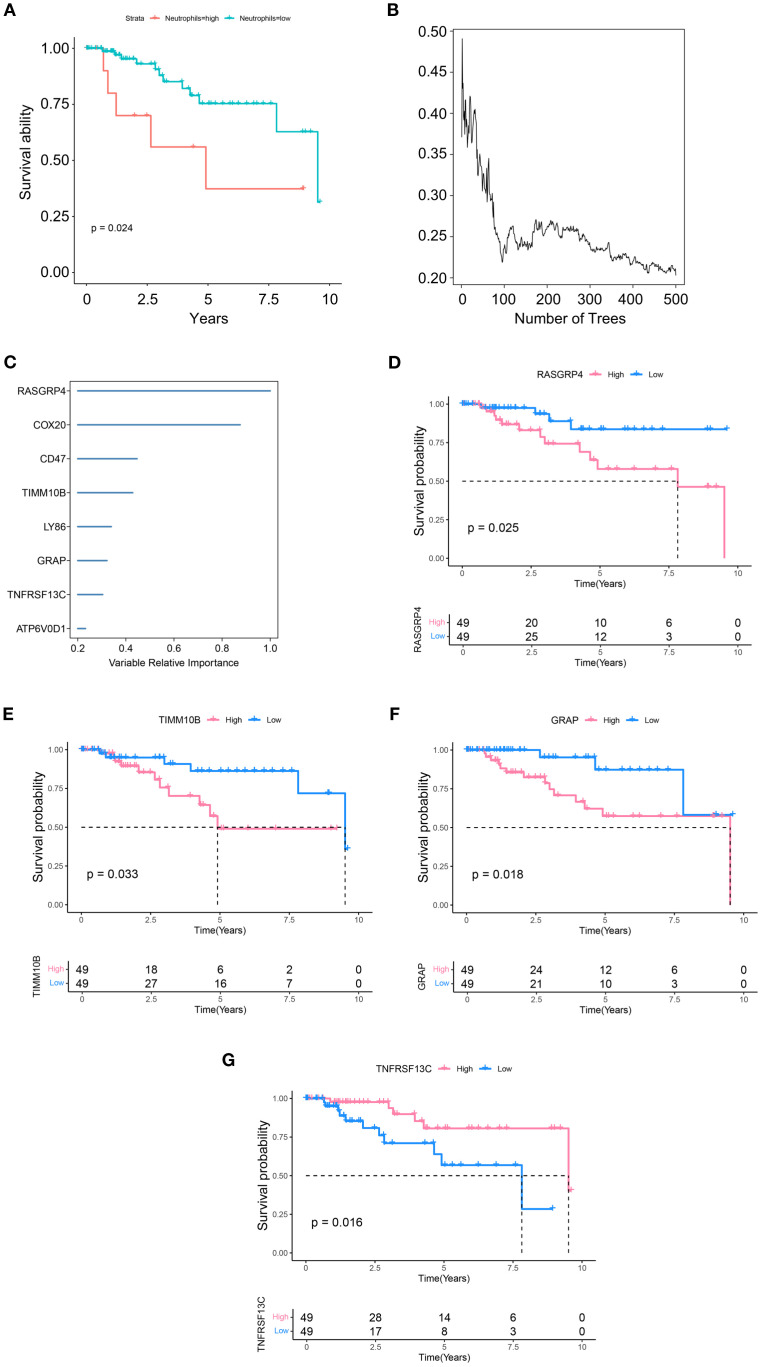
Identification of candidate genes. **(A)** Differences in survival between the high and low neutrophil group. **(B)** RSF analysis and expression levels of genes related to prognosis. **(C)** Expression levels and distributions of RASGRP4, COX20, CD47, TIMM10B, LY86, GRAP, TNFRSF13C and ATP6V0D1 in different tumor subtypes. **(D-G)** Kaplan–Meier graphs displaying the survival potential of patients with TNBC, grouped by the expression levels of significant genes.

To investigate the role of TANs in TNBC, we performed deconvolution analysis using the InstaPrism (v1.0.0) tool on bulk RNA-sequencing data from the TCGA-BRAC cohort. Patients were stratified into high and low neutrophil infiltration groups based on an optimal cutoff value determined by maximizing the survival difference between groups. The results showed that greater neutrophil infiltration was positively correlated with poorer patient survival (**Figure 2A**). We then utilized 1,436 marker genes from neutrophils for RSF analysis and identified those with a relative importance score exceeding 0.2 as final markers, thereby emphasizing the importance ranking of eight genes (**Figures 2B, C**). Subsequent survival analysis of these eight highly significant genes revealed that four genes, TIMM10B, GRAP, TNFRSF13C, and RASGRP4, were significantly associated with survival outcomes (p<0.05) (**Figures 2D–G**). Conversely, no significant differences were detected for COX20, CD47, LY86, or ATP6V0D1 (**Supplementary Figures S2A–D**).

### Immunoinfiltration patterns of the candidate genes

**Figure 3:**
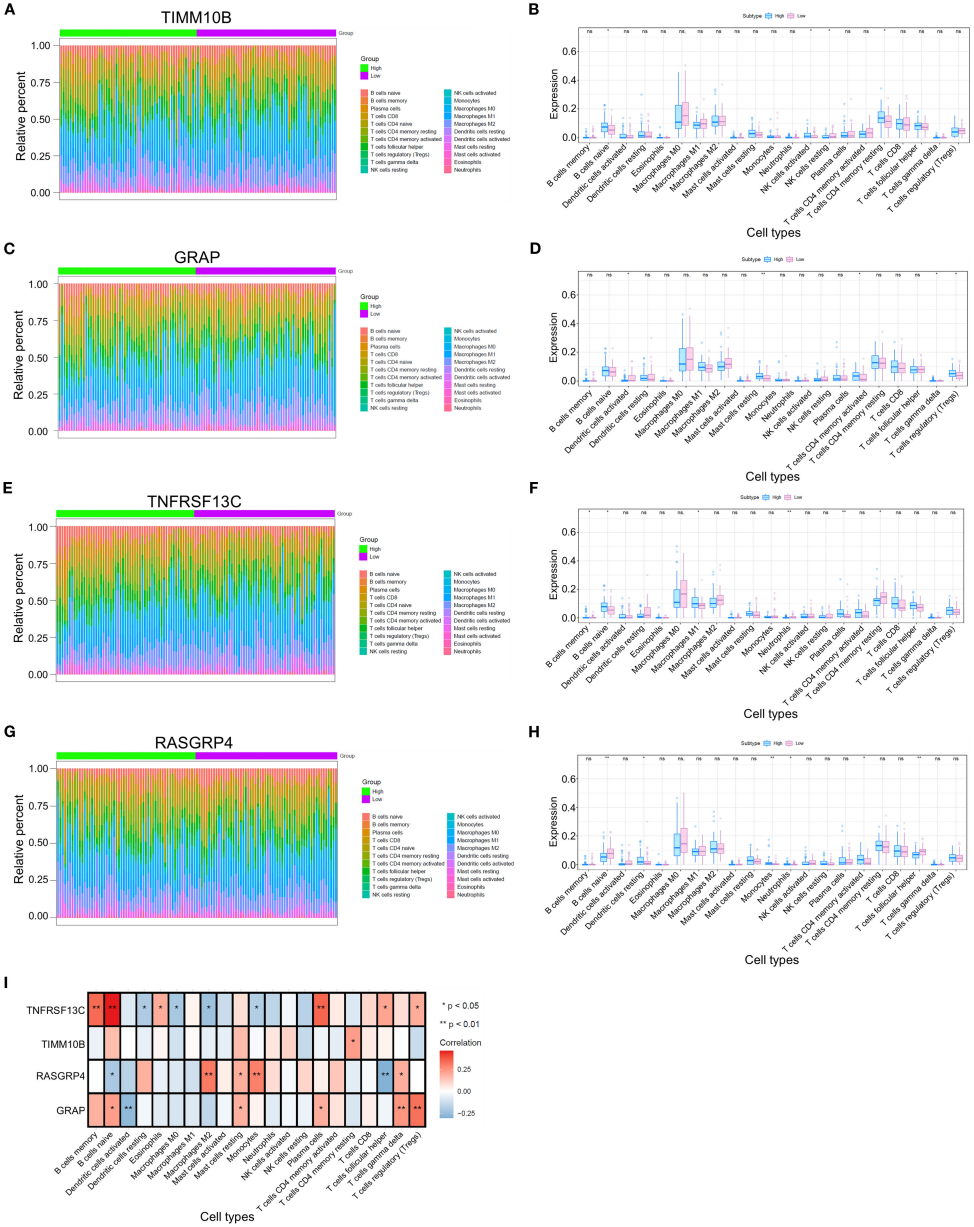
Differences in immune cell infiltration among the four different groups. **(A, C, E, G)** Heatmap illustrating the distributions of 22 immune cell subsets via ssGSEA.**(B, D, F, H)** Proportions of various types of infiltrating immune cells in the high- and low-risk groups. **(I)** Relevance of the key genes and immune cells. *p < 0.05 and **p < 0.01. ns, not significant.

Given that TANs are known to facilitate tumor progression and metastasis mainly through immunosuppressive effects, we further investigated the immune infiltration patterns associated with TAN-related gene expression. We then compared the proportions of immune cells between groups characterized by high or low expression of each key gene in various forms (**Figures 3A, C, E, G**). Significant differences in the proportions of naive B cells, activated NK cells, resting memory CD4 T cells, and resting NK cells were detected between groups with different TIMM10B expression levels (**Figure 3B**). In addition, there were marked differences in the proportions of resting mast cells, activated memory CD4 T cells, regulatory T cells (Tregs), activated dendritic cells, and gamma delta T cells between groups with high and low GRAP expression (**Figure 3D**). When comparing the groups with high and low TNFRSF13C expression, notable differences in the proportions of memory CD4 T cells, resting memory B cells, naive B cells, M1 macrophages, plasma cells, neutrophils, and T cells were detected between the two groups (**Figure 3F**). Similarly, notable differences between the groups with high and low RASGRP4 expression were found in the proportions of resting dendritic cells, follicular helper T cells, naive B cells, monocytes, activated memory CD4 T cells, and neutrophils (**Figure 3H**).

We conducted a comprehensive analysis of the associations between key genes and immune cell populations. Our findings revealed that TIMM10B was significantly positively correlated with resting memory CD4 T cells. Additionally, GRAP was positively correlated with naive B cells, resting mast cells, plasma cells, gamma delta T cells, and regulatory T cells (Tregs) but significantly negatively correlated with activated dendritic cells. Moreover, TNFRSF13C was significantly positively correlated with memory B cells, naive B cells, eosinophils, plasma cells, follicular helper T cells, and regulatory T cells (Tregs) and negatively correlated with resting dendritic cells, M0 macrophages, M2 macrophages, and monocytes. Conversely, RASGRP4 was positively correlated with M2 macrophages, resting mast cells, monocytes, and gamma delta T cells, and significantly negatively correlated with naive B cells and follicular helper T cells (**Figure 3I**).

Furthermore, we analyzed the associations between key genes and various immune factors, including immunosuppressors, immunostimulators, chemokines, and receptors. The results suggest that key genes are intricately associated with the degree of immune cell infiltration and play a significant role in modulating the immune microenvironment (**Supplementary Figures S3A–E**).

### Identification of potential drugs for TNBC patients

**Figure 4:**
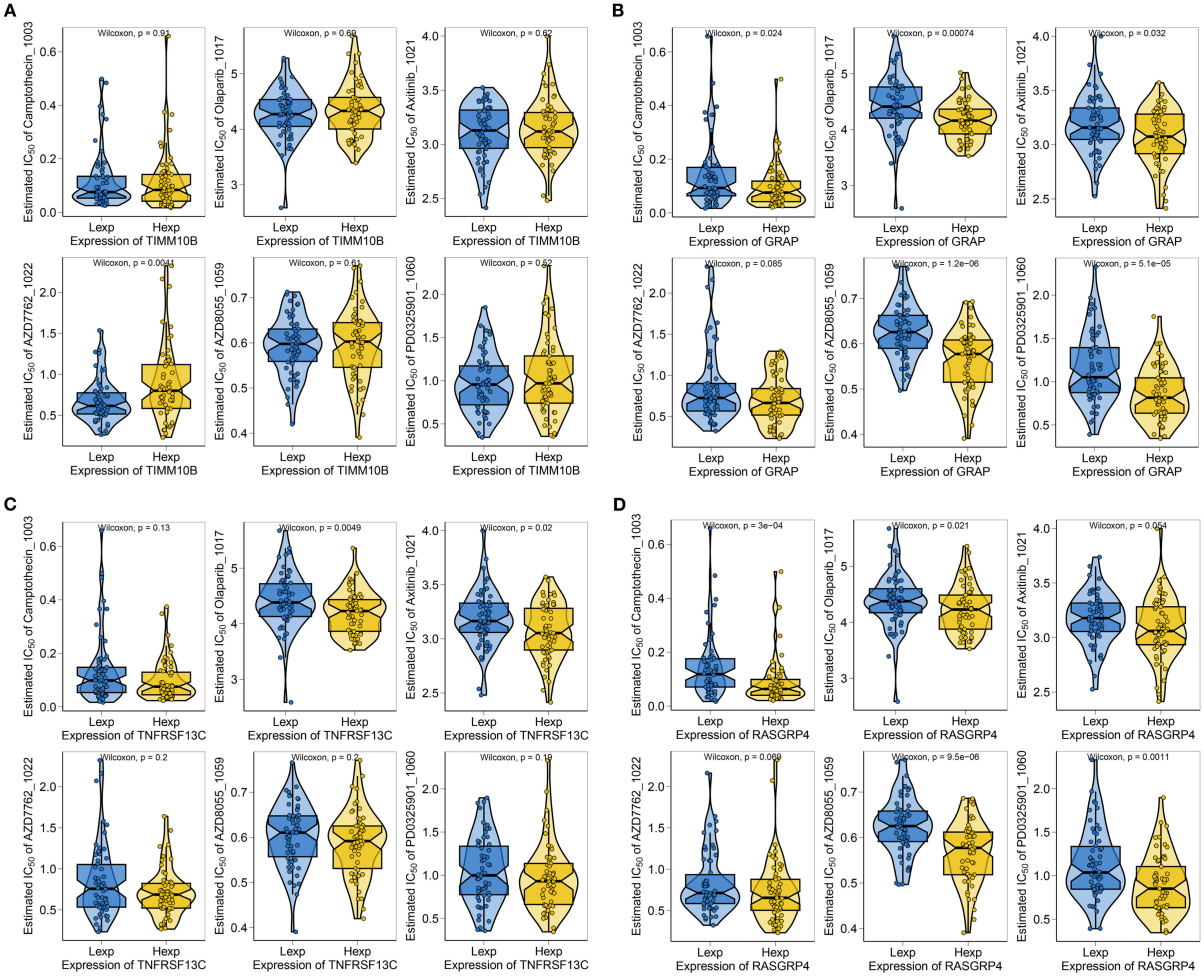
Correlations between TIMM10B, GRAP, TNFRSF13C and RASGRP4 and drug sensitivity. **(A-D)** Correlations between key genes and the IC50 of chemotherapeutic agents.

To further explore the clinical significance of the risk scores and prognostic genes, we determined the IC50 values for different drugs in TNBC patients. By leveraging drug sensitivity data from the GDSC database and applying the oncoPredict R package, we predicted the response of individual tumor samples to chemotherapy, with a focus on the TIMM10B, GRAP, TNFRSF13C, and RASGRP4 genes in relation to commonly used chemotherapeutic agents. The analysis revealed that TIMM10B was associated with sensitivity to AZD7762_1022 (**Figure 4A**). GRAP was associated with sensitivity to camptothecin_1003, olaparib_1017, axitinib_1021, AZD8055_1059, and PD0325901_1060 (**Figure 4B**). TNFRSF13C was associated with sensitivity to olaparib_1017 and axitinib_1021 (**Figure 4C**). RASGRP4 was associated with sensitivity to camptothecin_1003, olaparib_1017, AZD8055_1059, and PD0325901_1060 (**Figure 4D**). These findings highlight the potential of these biomarkers in predicting responsiveness to chemotherapy, thereby providing valuable insights for the development of personalized treatment strategies for patients with triple-negative breast cancer.

### GSEA and GSVA analysis

**Figure 5:**
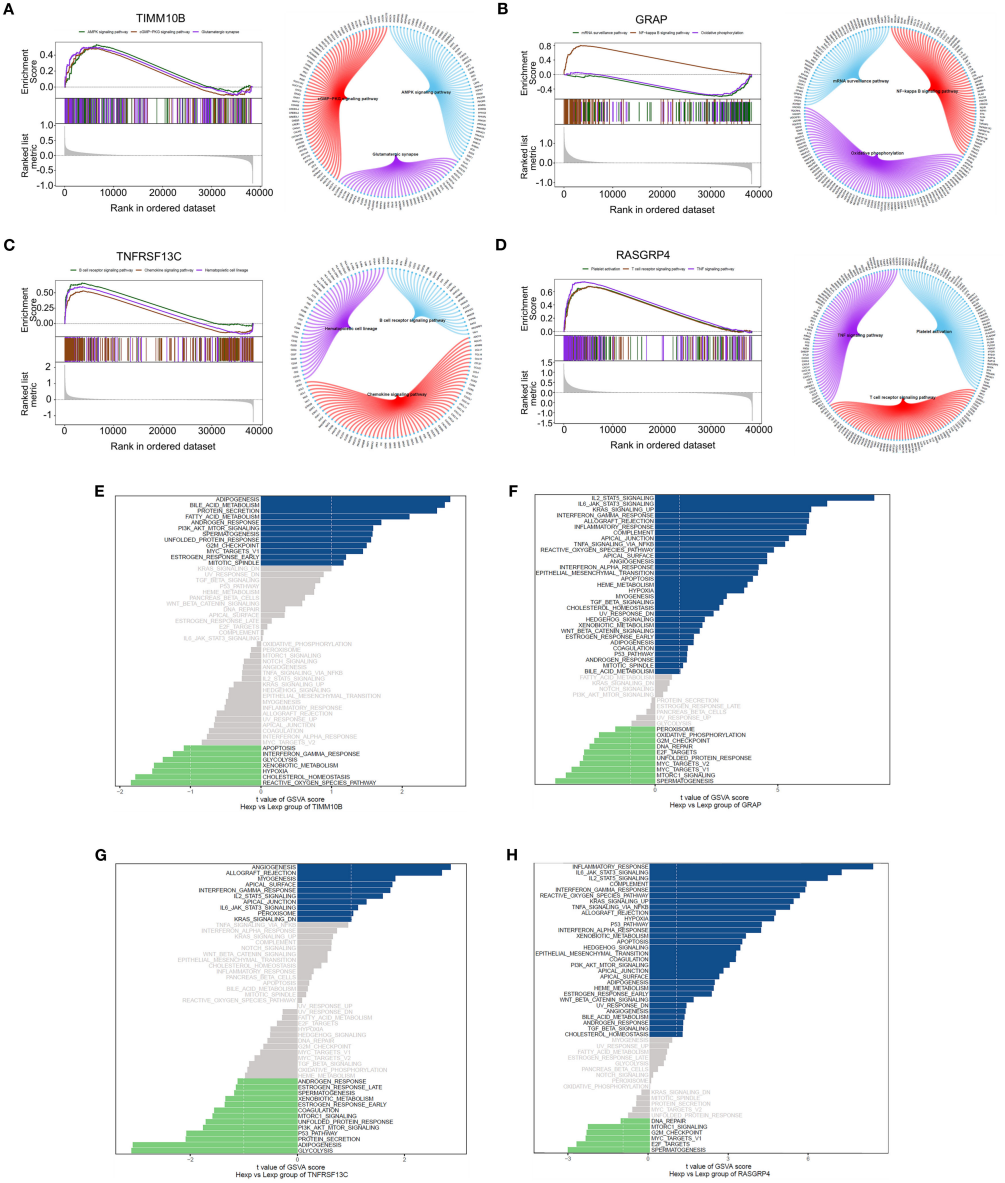
GSEA and GSVA of the four genes. **(A-D)**. GSEA revealed the enriched signaling pathways associated with TIMM10B, GRAP, TNFRSF13C and RASGRP4. **(E-H)**. Analysis of key genes using GSVA. The x-axis illustrates the t value of the GSVA score, and the y-axis depicts KEGG pathways; blue highlights upregulated pathways, whereas green highlights downregulated pathways. |NES| ≥ 1 and FDR < 0.25.

We then investigated the specific signaling pathways involving key genes and studied the molecular mechanisms through which these genes impact disease progression. GSEA revealed that TIMM10B was associated with the AMPK signaling pathway, cGMP-PKG signaling pathway, glutamatergic synapse, and other pathways (**Figure 5A**). GRAP was associated with the NF-kappa B signaling pathway, the mRNA surveillance pathway, and oxidative phosphorylation (**Figure 5B**). TNFRSF13C was strongly associated with B-cell receptor signaling pathway, chemokine signaling pathways, hematopoietic cell lineage pathways, and other signaling pathways (**Figure 5C**). RASGRP4 was prominently associated with signaling pathways, including the TNF signaling pathway, platelet activation, and the T-cell receptor signaling pathway (**Figure 5D**).

GSVA revealed that TIMM10B was associated with pathways linked to adipogenesis, bile acid metabolism, and various other signaling pathways (**Figure 5E**). GRAP was associated with IL2/STAT5 signaling, IL6/JAK/STAT3 signaling, and other signaling pathways (**Figure 5F**). TNFRSF13C was associated with angiogenesis, allograft rejection, and other signaling pathways (**Figure 5G**). RASGRP4 was associated with the inflammatory response and IL6/JAK/STAT3 signaling pathways (**Figure 5H**). These data suggest that these key genes could play a part in the progression of triple-negative breast cancer through these pathways.

### Assessment of the prognostic effect

**Figure 6:**
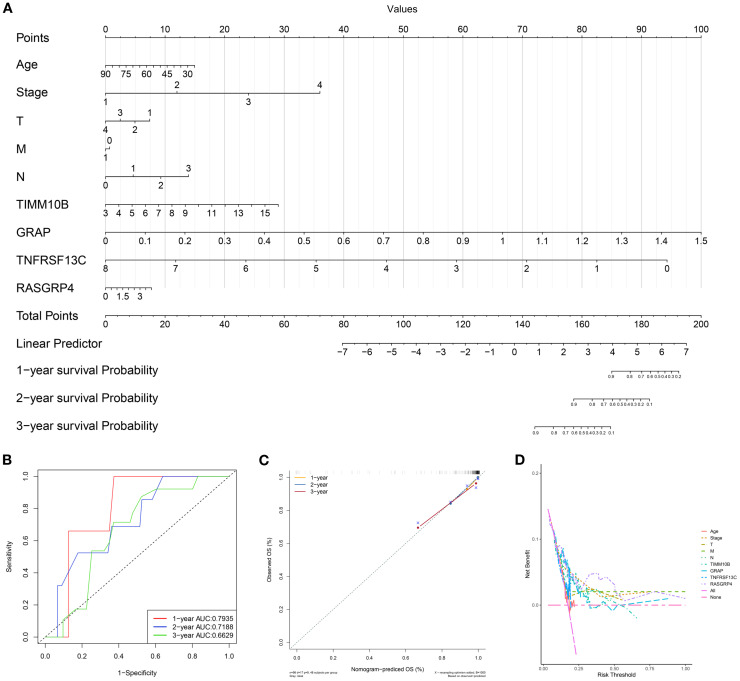
Establishment of the prognostic risk model. **(A)** Nomogram for predicting the survival of patients according to key genes. **(B)** A calibration curve of the nomogram was used to assess accuracy over 1, 3, and 5 years. **(C)** Multivariate ROC curve and risk score. **(D)** Evaluation of model performance using the concordance index. Abbreviations: T, tumor stage; M, metastasis stage; N, node stage; OS, overall survival; AUC, area under the curve.

Based on the multivariate Cox regression coefficients of the gene signature and clinical traits (age and TNM stage) of patients in the TCGA training dataset, we built a prognostic nomogram for clinicians to quantitatively predict the 1-, 3- and 5-year OS probabilities of TNBC patients. The outcomes of the regression analysis were presented in a bar graph, reflecting the expression levels of pivotal genes. This analysis demonstrated that the values of diverse clinical indicators and the distribution of key gene expression among all samples contributed variably to the overall scoring process (**Figure 6A**). Additionally, a predictive analysis of overall survival (OS) data for one, three, and five years was conducted (**Figures 6B–D**). The findings demonstrated that the predicted OS was closely aligned with the observed OS data, confirming the efficiency of the nomogram model.

### Cell communication and quasitemporal analysis

**Figure 7:**
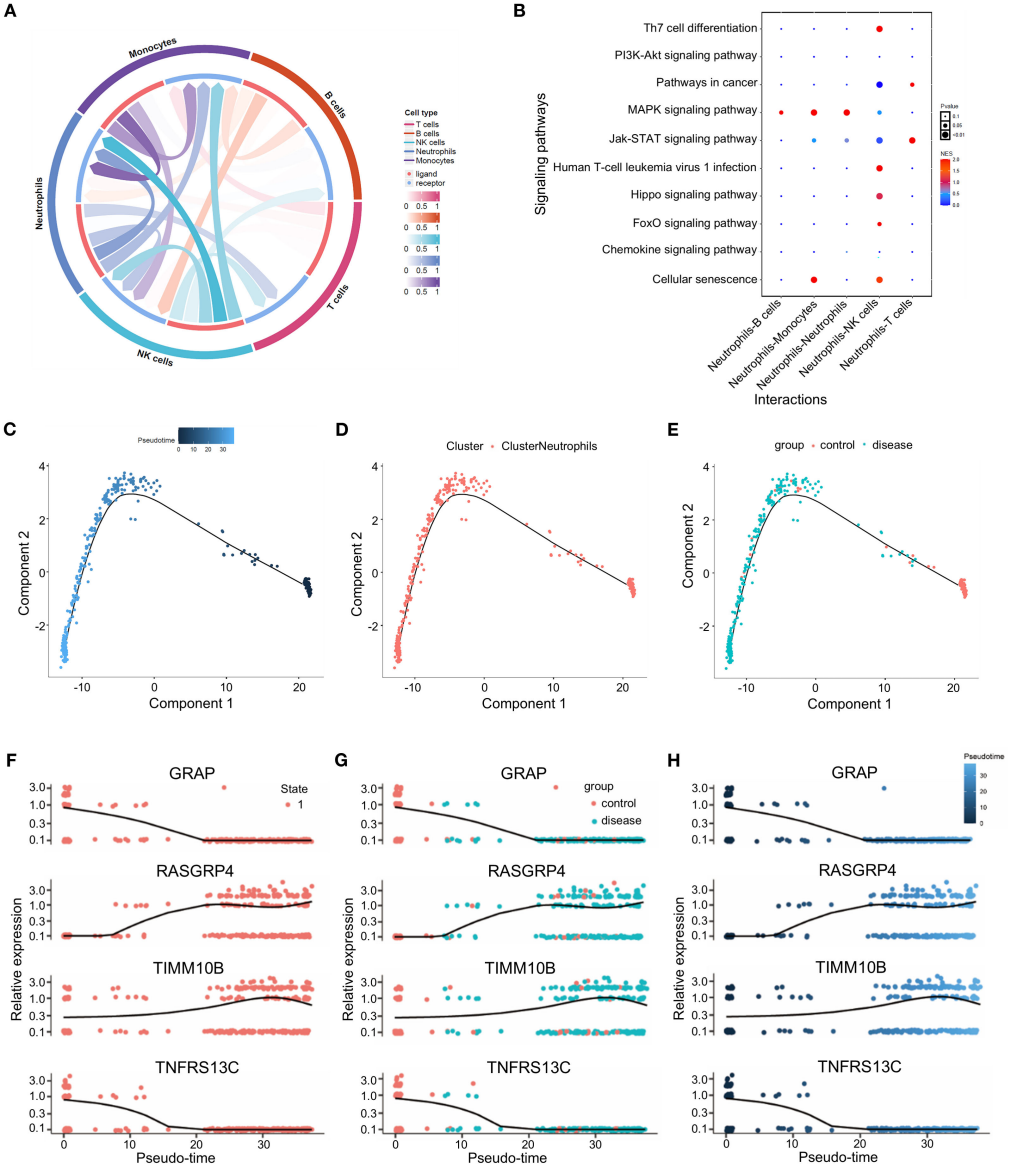
Cell communication and quasitemporal analysis. **(A)** Circus plot illustrating the greater total number of significantly interacting pairs between neutrophils and immune cells as estimated by CellPhoneDB (P<0.05). **(B)** Bubble diagram of the cell communication network between ligands and neutrophils and other cell subtypes as well as with neutrophil itself themselves. **(C-E)** Trajectory analysis of the potential relatedness between the two groups according to pseudotime, cell type and group. **(F-H)** Changes in the expression of TIMM10B, GRAP, TNFRSF13C and RASGRP4 over pseudotime.

To explore the interplay between neutrophils and other cells, we analyzed the ligand–receptor relationships of features in the single–cell expression profile of triple-negative breast cancer. We found complex interaction pairs among these cell types (**Figure 7A**), and demonstrated that ligands of neutrophils interact with other cell types as well as with neutrophils themselves (**Figure 7B**). Next, a quasitemporal analysis was conducted, where we initially determined the similarity between cells and built cell differentiation pathways. By visualizing the trajectory, a pseudotime picture of cell differentiation was constructed to illustrate the cell development process, which is useful for studying cell differentiation and gene expression. Illustrations showing cell coloring with pseudotime values, cell types, and groups were produced individually (**Figures 7C–E**). The changes in the expression of key genes from the start to the end of the pseudotime process are shown in **Figures 7F–H**.

### Overview of key genes in single cells

**Figure 8:**
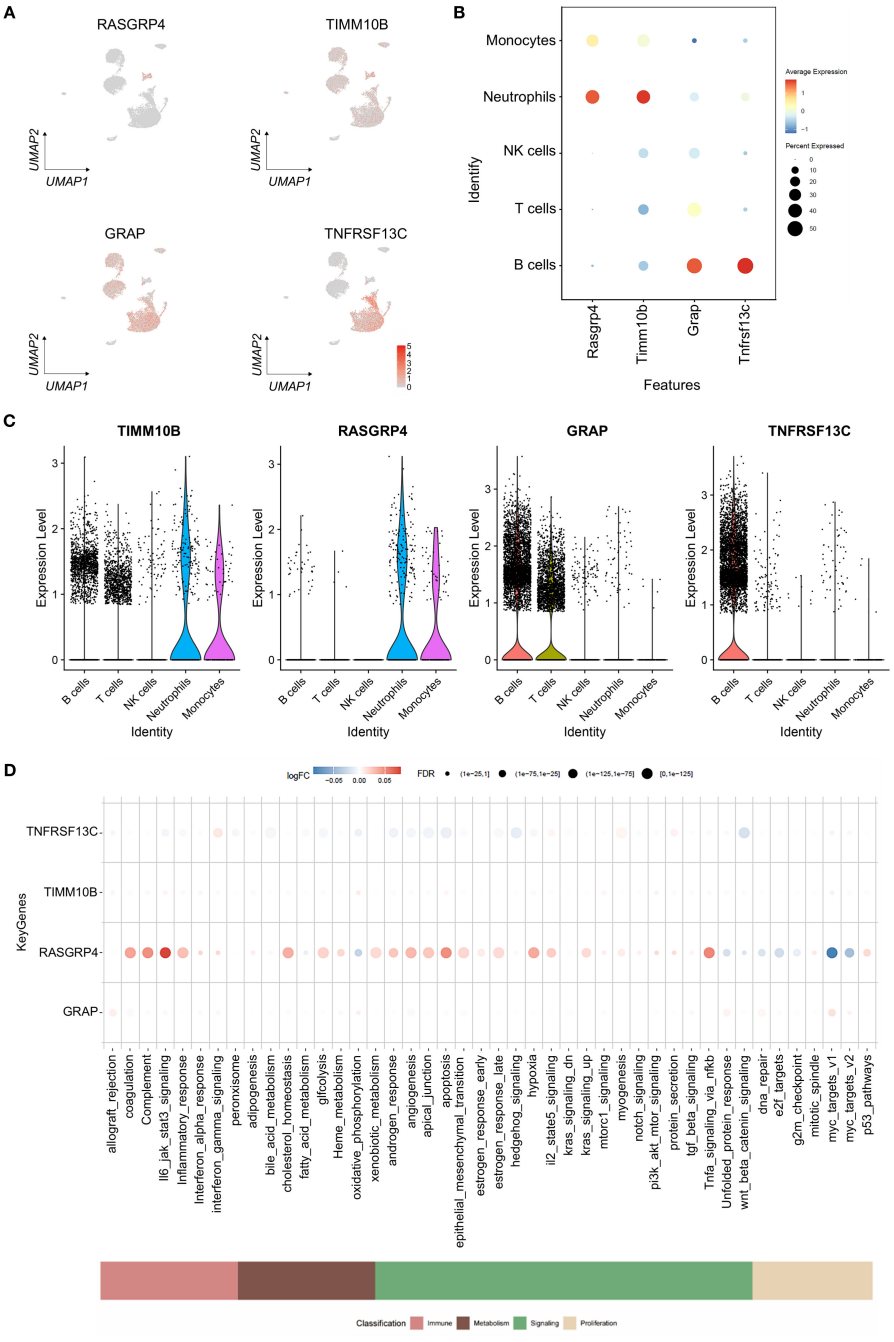
Gene expression levels in cells. **(A)** Atlas showing the expression levels of the specified genes, with red denoting expression. **(B)** Bubble plot showing the expression levels of the selected genes in different cell types. **(C)** Violin plots illustrating the expression levels of specific genes that define the inferred cell types. **(D)** Bubble plot illustrating variations in the activity of key genes involved in immune metabolism pathways.

To analyze the expression of crucial genes in single cells, we employed the FeaturePlot and Dotplot functions from the SeuratR package (**Figures 8A–D**). The Results showed that RASGRP4 and TIMM10B were highly expressed in neutrophils, while GRAP and TNFRSF13C were down-regulated in neutrophils (**Figures 8A–C**). We also checked the expression pattern of the four targeted genes in tumor-free and TNBC-bearing mice. The results revealed that RASGRP4 and TIMM10B were highly expressed in TNBC-bearing mice, while GRAP and TNFRSF13C were highly expressed in tumor-free mice (**Supplementary Figure S4**). AUCell was used to quantitatively score genes related to immune metabolism in single cells, and bubble map was generated to visualize the differences in the activity of key genes in immune metabolism-related pathways. The results showed high activity of Tnfrsf13c in interferon_gamma_response and other pathways; high activity of Rasgrp4 is in il6_jak_stat3_signaling; and tnfa_signaling_via_nfkb and other pathways; and high activity of Grap in allograft_rejection, myc_target_v1 and other pathways (**Figure 8D**).

### RASGRP4 and TIMM10B exhibit pro-tumor activities

**Figure 9:**
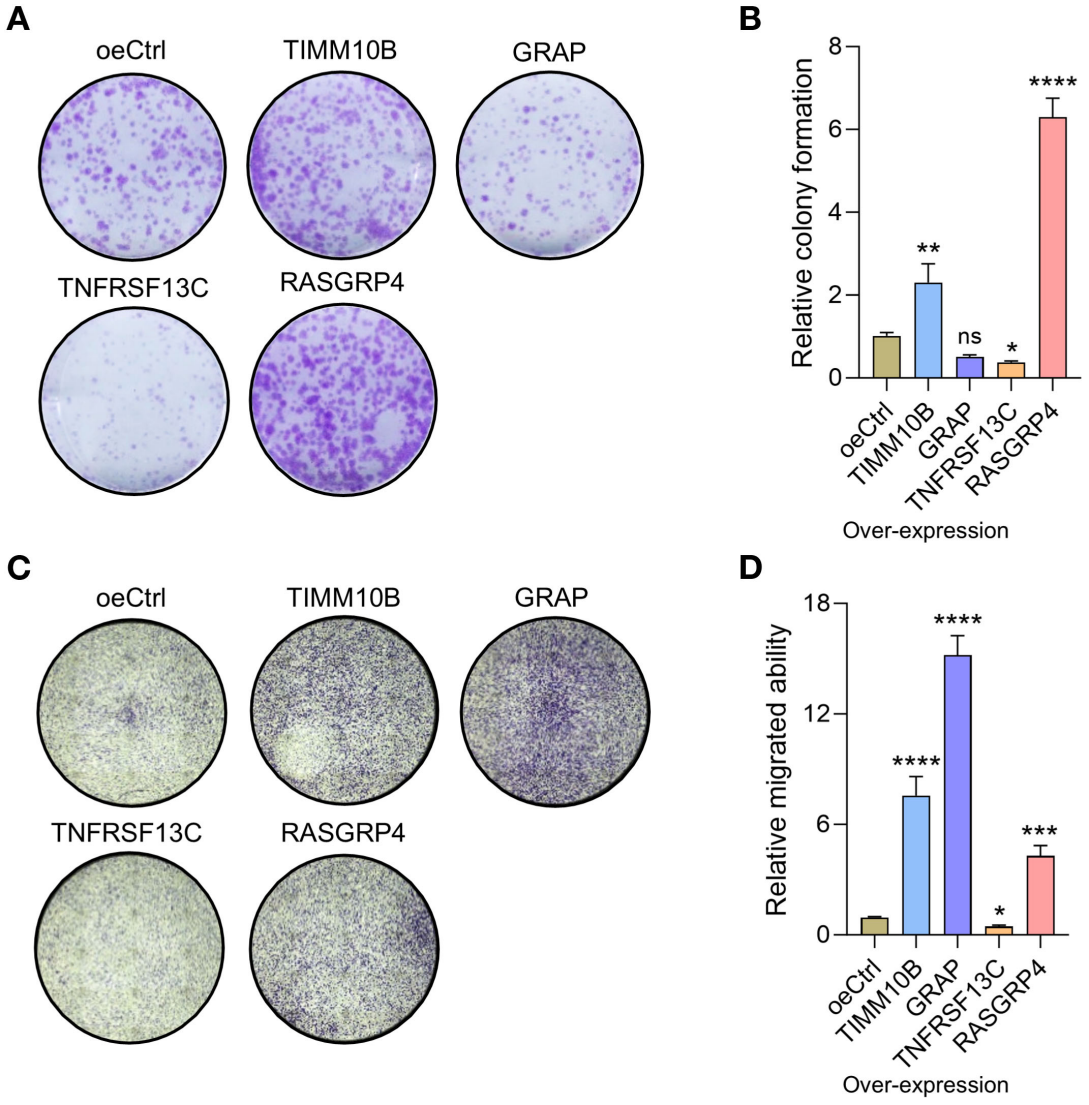
RASGRP4, TIMM10B and TNFRSF13C exhibited protumorigenic activities. **(A)** Representative images of colonies of MDA-MB-231 cells treated with control or gene-overexpressing neutrophils. **(B)** Relative quantified number of colonies. **(C)** Representative images of migrated cells after cocultured with control or gene-overexpressing cells. **(D)** Quantification of the relative number of migrated cells.

To evaluate the influence of the four candidate genes on the pro-tumor functions of neutrophils, we overexpressed these genes in dHL-60 cells. The qRT-PCR results confirmed the significant overexpression of all four genes (**Supplementary Figure S4A**). In the colony formation assay, MDA-MB-231 cells cultured with supernatants from RASGRP4- and TIMM10B-overexpressing dHL-60 cell cultures presented increased colony formation ability. In contrast, MDA-MB-231 cells cultured with supernatants from TNFRSF13C-overexpressing dHL-60 cell cultures showed a reduced colony formation capacity, whereas no significant difference was observed in MDA-MB-231 cells cocultured with GRAP-overexpressing dHL-60 cell culture supernatants (**Figures 9A, B**). Additionally, Transwell migration assays revealed that compared with control cells, MDA-MB-231 cells cocultured with RASGRP4, TIMM10B, or GRAP-overexpressing dHL-60 cells exhibited increased migration ability (**Figures 9C, D**). We also cocultured dHL-60 cells with MDA-MB-231 cells and then examined the mRNA levels of the four genes. The mRNA levels of TIMM10B and RASGRP4 were significantly increased, whereas the level of TNFRSC13C was decreased. There was no change in the mRNA level of GRAP (**Supplementary Figure S4B**).

These findings imply that a neutrophil subpopulation with upregulated RASGRP4 and TIMM10B expression and downregulated TNFRSF13C may contribute to pro-tumor activities within the tumor microenvironment.

### Elevated RASGRP4 and TIMM10B expression, alongside reduced TNFRSF13C expression

**Figure 10:**
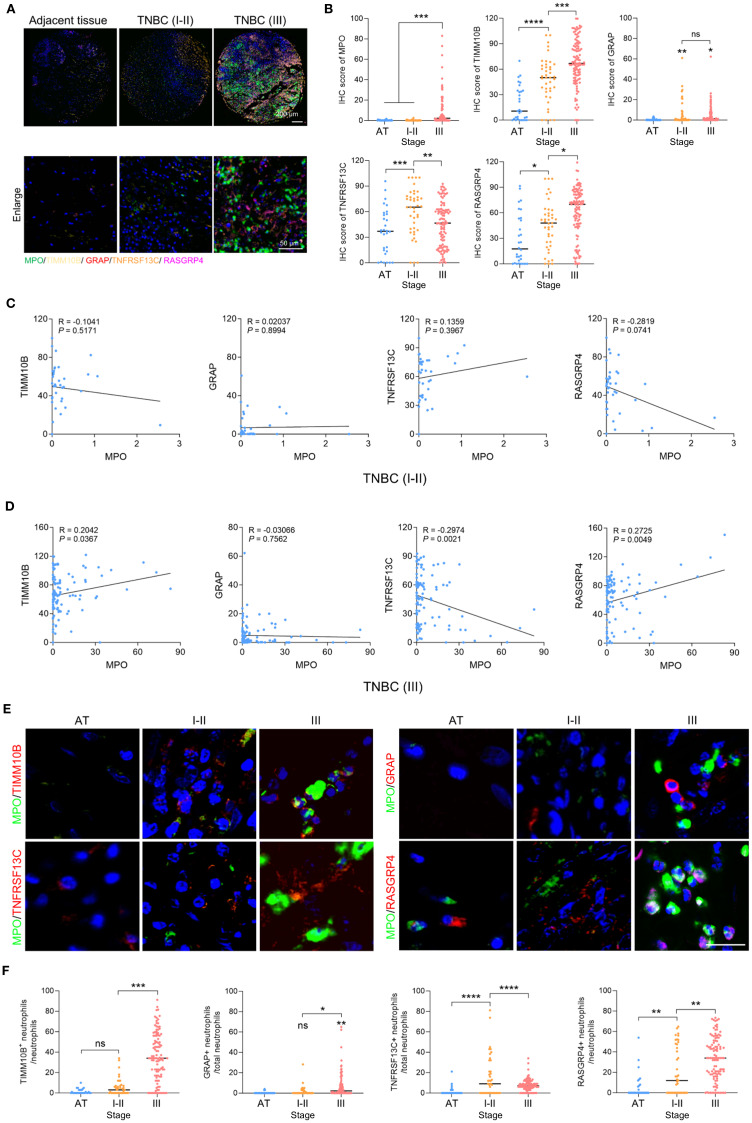
Clinical relevance of TIMM10B, GRAP, TNFRSF13C and RASGRP4. **(A)** Polychromatic immunofluorescence staining showing the distribution of MPO, TIMM10B, GRAP, TNFRSF13C and RASGRP4 expression. Scale bar (upper panel), 200 µm. Scale bar (bottom panel), 50 µm. **(B)** IHC scores of MPO, TIMM10B, GRAP, TNFRSF13C and RASGRP4 in adjacent tissue (AT), stage I-II **(I-II)** and III TNBC. **(C)** Correlations between MPO and the four candidate genes in stage I-II **(I-II)** TNBC. **(D)** Correlations between MPO and the four candidate genes in stage III **(III)** TNBC. **(E)** Representative images of coimmunostaining for MPO and four target genes in adjacent tissue from the stage I-II and III TNBC groups. Scale bar, 20 µm. **(F)** Percentage of TIMM10B-, GRAP-, TNFRSF13C- and RASGRP4-positive cells in AT and stage I-II and III TNBC. *p < 0.05, **p < 0.01, ***p < 0.001, ****p < 0.0001. ns, not significant.

To confirm the clinical importance of these newly identified candidate genes, we utilized polychromatic immunofluorescence staining to evaluate the expression levels of the neutrophil marker MPO and the genes TIMM10B, GRAP, RASGRP4, and TNFRSF13C in TNBC (**Figure 10A**). The results revealed that MPO, TIMM10B, and RASGRP4 were expressed at significantly higher levels in grade III TNBC tumors than in both adjacent tissues and grade I–II tumors, whereas TNFRSF13C expression was lower in grade III tumors than in grade I–II tumors (**Figure 10B**). There was no notable difference in GRAP expression between grade I–II and grade III TNBC tumors (**Figure 10B**).

Next, we analyzed the correlation between MPO and these four target genes. In grade I–II TNBC tumors, there was no correlation between MPO and TIMM10B, GRAP, or TNFRSF13C, but MPO was negatively correlated with RASGRP4 (**Figure 10C**). In contrast, in grade III TNBC tumors, MPO was positively correlated with TIMM10B and RASGRP4 but negatively correlated with TNFRSF13C (**Figure 10D**). Moreover, neutrophils expressing TIMM10B and RASGRP4 were more frequently observed in stage III TNBC than in stage I–II TNBC and normal tissues, whereas neutrophils expressing TNFRSF13C were less frequently observed in stage III TNBC than in stage I–II TNBC (**Figures 10E, F**). Taken together, these findings suggest that higher expression of TIMM10B and RASGRP4 in grade III TNBC tumors is associated with MPO expression and a poor prognosis, whereas lower TNFRSF13C expression in grade III TNBC tumors is negatively correlated with MPO expression.

## Discussions

TANs are integral to the TME of TNBC, where they exhibit both tumor-promoting and immune-modulating functions (29). In our study, we performed a detailed single-cell transcriptomic analysis of the TME in TNBC, which led to the identification of crucial prognostic genes—TIMM10B, RASGRP4, and TNFRSF13C—that demonstrate significant clinical relevance in TNBC patients. The predictive accuracy of these genes was validated using a nomogram model, underscoring their potential as biomarkers for patient prognosis. These results align with those of previous studies linking TANs to cancer aggressiveness through mechanisms such as the formation of neutrophil extracellular traps (NETs) and the inhibition of cytotoxic T lymphocytes (CTLs) (30–32).

The analysis of immune infiltration demonstrated that these genes are intricately associated with various immune cell populations. For example, GRAP was positively correlated with Tregs and negatively correlated with activated dendritic cells, indicating its role in fostering an immunosuppressive microenvironment. Similarly, TIMM10B and RASGRP4 were significantly correlated with macrophages and T cells, highlighting their involvement in modulating the immune response within the TME. These interactions reflect mechanisms by which neutrophils suppress cytotoxic T lymphocytes, thereby facilitating tumor progression and metastasis. Notably, TIMM10B, RASGRP4, and GRAP were linked to poorer survival outcomes, suggesting their participation in tumor-promoting pathways, whereas TNFRSF13C exhibited context-dependent roles that may influence immune modulation within the TME. Although several studies have investigated the inflammatory response and immunomodulatory roles of GRAP, TNFRSF13C, and RASGRP4 (33–37), few investigations have focused on the immunomodulatory functions of these four genes in neutrophils specifically.

GSEA and GSVA revealed that the four candidate genes are involved in diverse signaling pathways, suggesting their potential roles in regulating TNBC progression through multiple biological mechanisms. These pathway associations support the notion that these genes may influence the tumor microenvironment via metabolic regulation, inflammation, and immune cell interactions, further reinforcing the multifaceted role of TANs in shaping the immune landscape in TNBC. However, we acknowledge that these pathway analyses are based on transcriptomic correlations and do not provide direct mechanistic evidence. Functional validation experiments—such as pathway inhibition assays, downstream target analysis, or reporter-based readouts—were not performed in the current study. This remains a key limitation. In future work, we aim to conduct mechanistic investigations using immune-tumor coculture systems, phospho-protein analysis, and pathway-specific perturbation models, to clarify the causal relationships between these genes and their downstream signaling effects in the tumor microenvironment of TNBC.

Furthermore, we assessed the sensitivity of these genes to chemotherapeutic agents via the GDSC database. TIMM10B, GRAP, TNFRSF13C and RASGRP4 were found to be associated with sensitivity to specific chemotherapies, suggesting their potential as biomarkers for predicting responsiveness to chemotherapy. These findings underscore the possibility of utilizing these genes to guide personalized treatment strategies for TNBC patients. For example, pharmacological inhibition of enzymes such as arachidonate 5-lipoxygenase (Alox5) has been shown to effectively eliminate neutrophil prometastatic activity and reduce metastasis (38). Similarly, targeting cathepsin C (CTSC) with compounds such as AZD7986 suppresses neutrophil recruitment and lung metastasis in breast cancer models (16). Our identification of TIMM10B, GRAP, TNFRSF13C and RASGRP4 as key genes further expands the potential therapeutic targets within TANs, offering promising avenues for disrupting protumorigenic interactions within the TME. Targeting TANs has already shown promise in preclinical models by reducing metastasis and modulating neutrophil functions.

Functional assays demonstrated that TIMM10B, GRAP, and RASGRP4 exerted protumorigenic effects, as their overexpression in neutrophil-like cells significantly enhanced both the colony formation and migration of TNBC cells. In contrast, the overexpression of TNFRSF13C resulted in a reduction in colony formation and migration, indicating its potential role as a tumor suppressor under certain conditions. While GRAP and RASGRP4 have been previously implicated in oncogenesis (39–41), TNFRSF13C has recently been identified as a new prognostic biomarker for cervical cancer (42), and the role of TIMM10B in cancer remains largely unexplored. These findings emphasize the complex, context-dependent functions of these genes in the biology of TNBC and highlight the necessity for therapeutic strategies that meticulously consider the immune context of tumors.

We found that the expression of the neutrophil marker MPO was positively correlated with TIMM10B and RASGRP4, indicating that the expression levels of these genes are closely associated with neutrophil infiltration, particularly in high-grade tumors. These findings support the theory that neutrophils are pivotal in determining the immune landscape in TNBC and accelerating tumor advancement. Additionally, the negative correlation between MPO expression and both GRAP and TNFRSF13C suggests that reduced expression of these genes in neutrophils may contribute to immune evasion in aggressive tumors. While GRAP and TNFRSF13C are expressed at relatively high levels in B cells and are not exclusively associated with neutrophils, it is important to clarify that our identification of neutrophil-associated marker genes was based on differential expression patterns specifically from neutrophil subclusters. This approach focused on genes that were either upregulated or downregulated within distinct neutrophil populations. Both GRAP and TNFRSF13C were selected because they were significantly downregulated in a specific neutrophil subpopulation, making them useful for identifying functionally distinct neutrophil states rather than serving as universal neutrophil markers. We believe that the prognostic associations observed for these genes reflect the functional heterogeneity within the neutrophil compartment, rather than simply the presence or abundance of neutrophils. Therefore, despite its downregulation in aggressive tumors, TNFRSF13C could serve as a valuable therapeutic target in settings where immune responses are more pronounced or responsive.

This validation in tissue samples is in line with accumulating clinical evidence showing that peripheral neutrophil characteristics can reflect systemic and local inflammation in cancer. For example, the neutrophil-to-lymphocyte ratio (NLR), a simple metric calculated from routine peripheral blood tests, has been reported to correlate with cancer-related inflammation and predict clinical outcomes across various malignancies (43, 44). These findings suggest that neutrophil activation in the circulation may mirror or even contribute to the inflammatory milieu within the tumor microenvironment. However, due to the limited availability of high-quality blood samples from TNBC patients—particularly those with sufficient circulating neutrophils—we were unable to directly validate the expression of these four genes in blood-derived neutrophils. Future studies incorporating matched blood and tumor tissue from the same patients will be critical to confirm the consistency of neutrophil activation across compartments and further elucidate its prognostic and mechanistic implications in TNBC.

Despite its promising results, this study is limited by its retrospective design and reliance on publicly available datasets, which may introduce biases. We fully acknowledge the importance of generalizability and actively sought additional publicly available TNBC scRNA-seq datasets. However, high-quality datasets derived from primary TNBC tumors with comparable tissue origins, processing protocols, and sufficient neutrophil representations are scarce. Integrating datasets with substantial batch effects or inconsistent tissue handling could introduce unwanted noise—particularly for neutrophil-related analyses, which are highly sensitive to sample processing. Despite the small sample size, GSE222854 comprises 12,316 high-quality single cells, enabling robust clustering and downstream analyses. Importantly, this dataset includes well-annotated tumor-associated neutrophils (TANs), which are central to our study. We plan to perform integrative validation as more suitable datasets become available.

While our functional validations provided initial insights, they were performed exclusively *in vitro* and focused primarily on tumor cell–intrinsic phenotypes such as proliferation, colony formation, and migration. These assays, however, do not capture potential immunoregulatory functions of the candidate genes within the tumor microenvironment (TME). Given the observed associations between gene expression and distinct immune infiltration patterns, particularly involving neutrophils, it is critical to explore whether these genes influence immune cell recruitment, activation, or suppression.

Future studies should incorporate immune–tumor coculture systems, cytokine profiling, and immune cell functional assays to assess the immunomodulatory roles of these genes. In addition, the use of advanced *in vivo* models—such as humanized mouse models or syngeneic tumor systems—will be essential to validate their impact on tumor–immune interactions under physiologically relevant conditions. Expanding the analysis to include more diverse and clinically representative patient cohorts will also increase the generalizability and translational value of our findings.

Our study specifically focused on CD45^+^ Ter119^-^ immune cells to investigate the roles of tumor-associated neutrophils and related immune populations in the tumor microenvironment. While this approach allows us to characterize immune cell-specific transcriptional dynamics with high resolution, it inherently excludes non-immune cellular components such as stromal cells, endothelial cells, and erythroid lineage cells. These non-immune cells are known to contribute significantly to tumor progression and patient prognosis, and their interactions with immune cells may influence the tumor ecosystem in complex ways. We acknowledge that restricting the analysis to CD45^+^ Ter119^-^ cells may introduce bias and limit the comprehensiveness of our findings. Future studies incorporating a broader range of cellular compartments, including non-immune cells, will be essential to fully elucidate the intricate cellular crosstalk and heterogeneity within the tumor microenvironment.

## Conclusions

In conclusion, higher expression levels of TIMM10B and RASGRP4 were associated with poorer patient survival and protumor functions, while lower expression of TNFRSF13C also correlated with worse prognosis and protumor activity. In contrast, the clinical relevance and functional role of GRAP remained ambiguous, as it was lowly expressed in neutrophils and showed limited impact in functional assays. Therefore, TIMM10B, RASGRP4, and TNFRSF13C may serve as valuable biomarkers for risk stratification and personalized therapy in TNBC patients. Their associations with immune infiltration, drug sensitivity, and tumor progression provide a strong rationale for further exploration as therapeutic targets.

Future research should focus on mechanistic studies to further elucidate how these genes influence TNBC progression and immune modulation, as well as clinical trials to validate their utility as therapeutic targets. Given the complexity of the TME in TNBC, incorporating both single-cell and bulk transcriptomic data will be essential for developing effective biomarkers and combination therapies targeting both cancer cells and the supportive TME.

## Data Availability

The data presented in the study are deposited in the GEO repository, accession number GSE222854.

## Supplementary material

Supplementary Figure 1 Single cell analysis of TNBC samples. **(A, B)** In total, 12,316 cells were identified. **(C)** Diagram presenting the disparities in gene expression levels among TNBC cells. Red dots indicate genes with high variability, and the black dots indicate genes with stable expression. **(D)** The top 50 principal components, with P-values less than 0.05, as determined by PCA. **(E, F)** The batch effect was eliminated by integrating and correcting the scRNA-seq data with the R package Harmony.

Supplementary Figure 2 Clinical relevance of COX20, CD47, LY86 and ATP6V0D1. **(A-D)** Kaplan-Meier plots illustrating the survival rates of TNBC patients based on the expression levels of crucial genes.

Supplementary Figure 3 The immune cell infiltration profiles of the four genes. **(A-E)** Bubble plot showing the state of immune cell infiltration.

Supplementary Figure 4 Expression patterns of the four selected genes in tumor-free and TNBC-bearing mice.

Supplementary Figure 5 The efficiency of RASGRP4, TIMM10B, TNFRSF13C and GRAP expression was examined by qRT-PCR. **(A)**. Quantified mRNA levels of TIMM10B, GRAP, TNFRSF13C and RASGRP4 after overexpressing these targeted genes in dHL-60 cells. **(B)**. Quantified mRNA levels of TIMM10B, GRAP, TNFRSF13C and RASGRP4 in dHL-60 cells after coculture with MDA-MB-231 cells for 3 days.
