# Supplementary material for: Identification of tumor associated neutrophils-related genes in triple-negative breast cancer for predicting prognosis and therapeutic response through integrated single-cell analysis
Source: Front Immunol. 2025 Sep 22;16:1613529. doi: 10.3389/fimmu.2025.1613529 (PMC12497826; doi:10.3389/fimmu.2025.1613529)
Supplement: Supplementary Figure 1 — Single cell analysis of TNBC samples. (A, B) In total, 12,316 cells were identified. (C) Diagram presenting the disparities in gene expression levels among TNBC cells. Red dots indicate genes with high variability, and the black dots indicate genes with stable expression. (D) The top 50 principal components, with P-values less than 0.05, as determined by PCA. (E, F) The batch effect was eliminated by integrating and correcting the scRNA-seq data with the R package Harmony. [file DataSheet1.docx]

**Supplementary figures**

**Fig. S1**


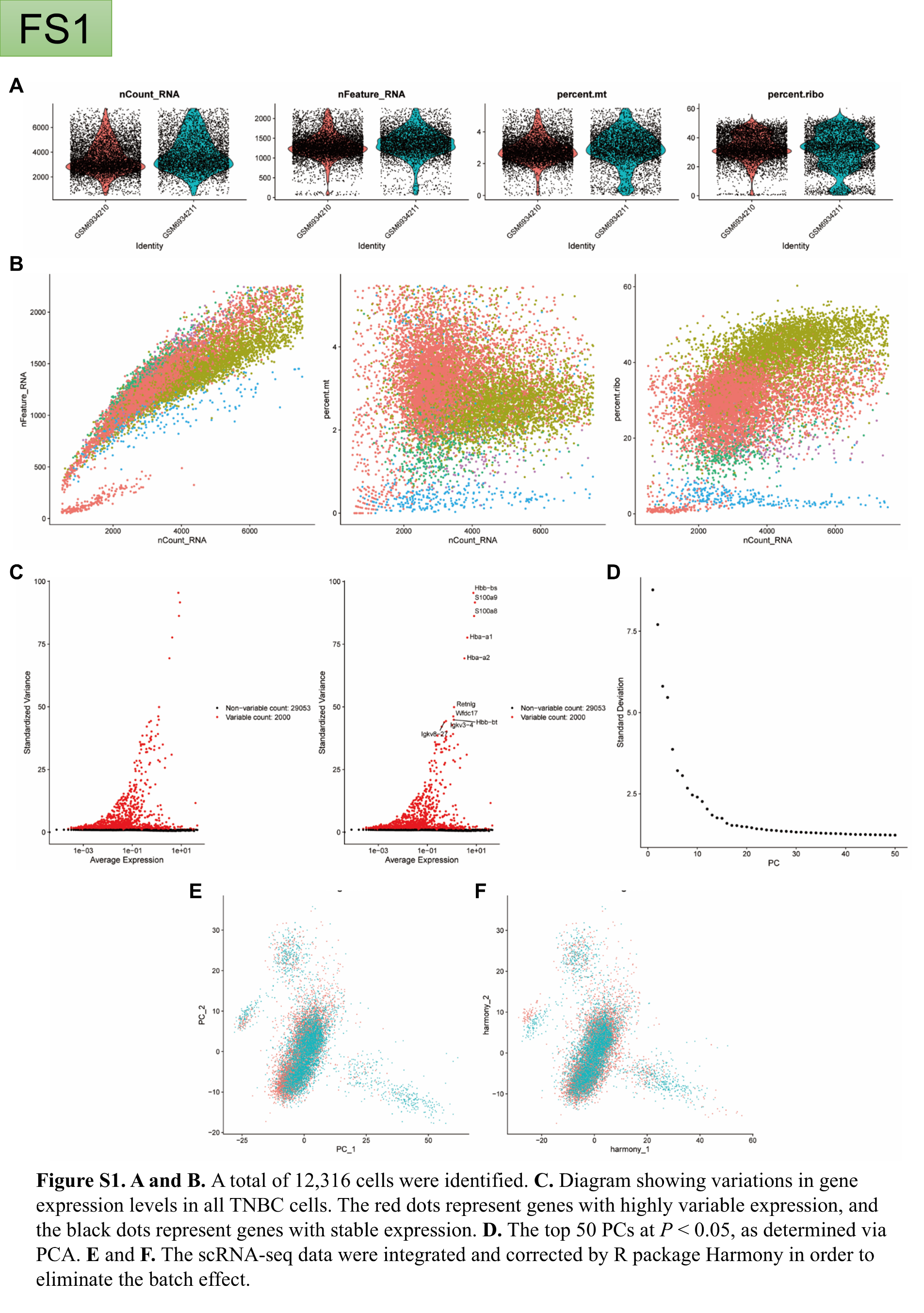


Single cell analysis of TNBC samples. (**A, B**) In total, 12,316 cells were identified. (**C**) Diagram presenting the disparities in gene expression levels among TNBC cells. Red dots indicate genes with high variability, and the black dots indicate genes with stable expression. (**D**) The top 50 principal components, with P-values less than 0.05, as determined by PCA. (**E, F**) The batch effect was eliminated by integrating and correcting the scRNA-seq data with the R package Harmony.

**Fig. S2**


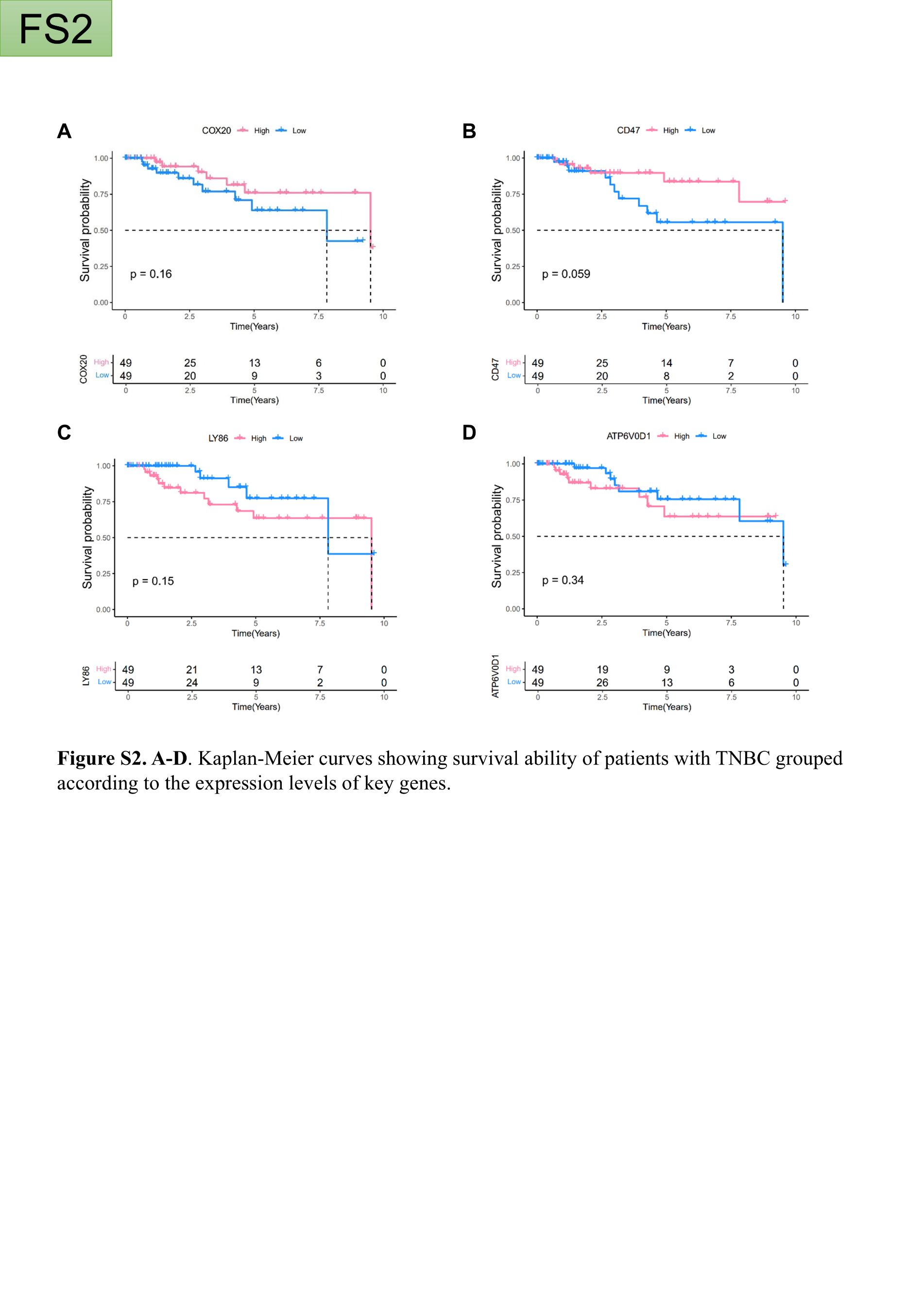


Clinical relevance of COX20, CD47, LY86 and ATP6V0D1. (**A-D**) Kaplan-Meier plots illustrating the survival rates of TNBC patients based on the expression levels of crucial genes.

**Fig. S3**


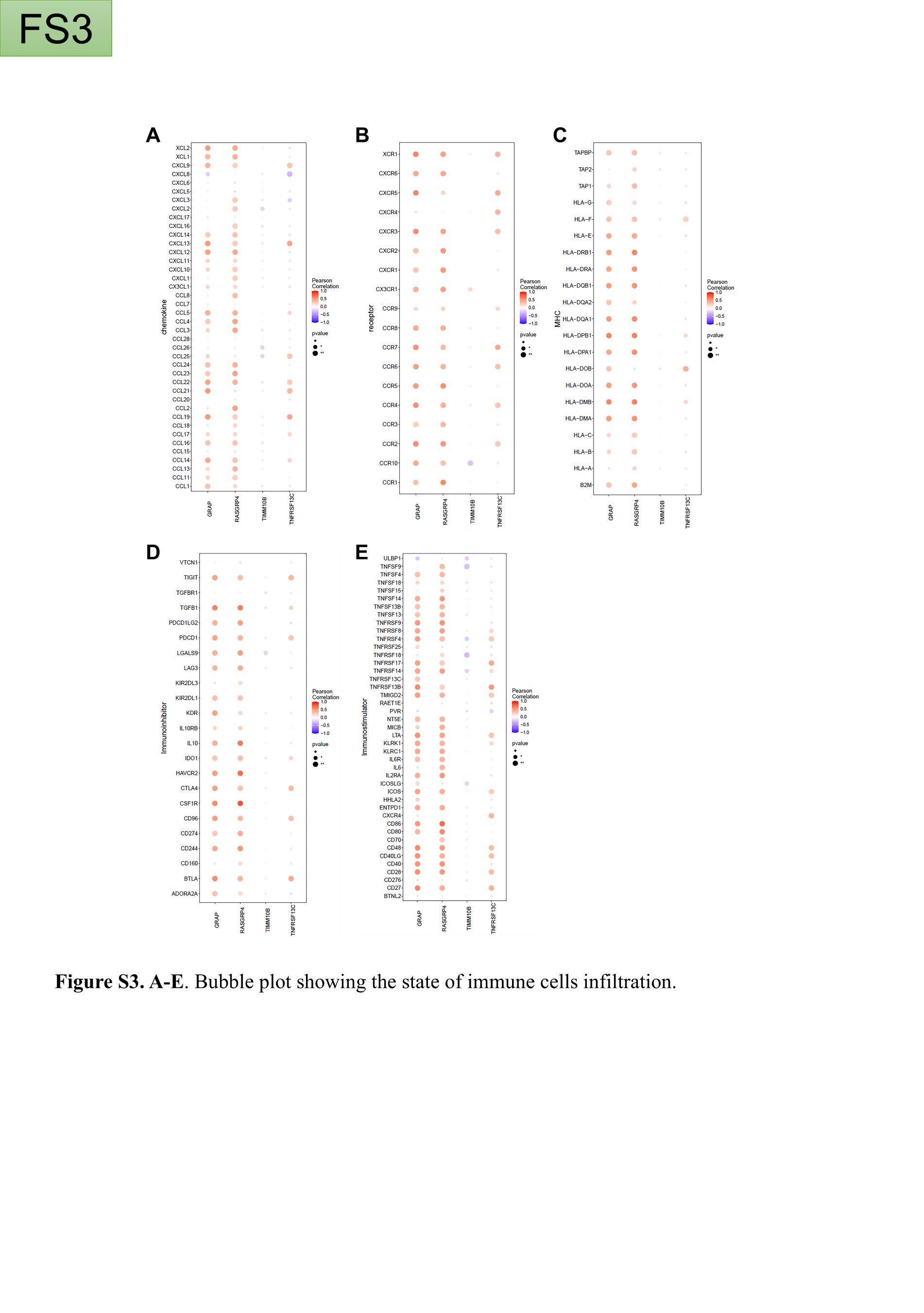


The immune cell infiltration profiles of the four genes. (**A-E**) Bubble plot showing the state of immune cell infiltration.

**Fig. S4**


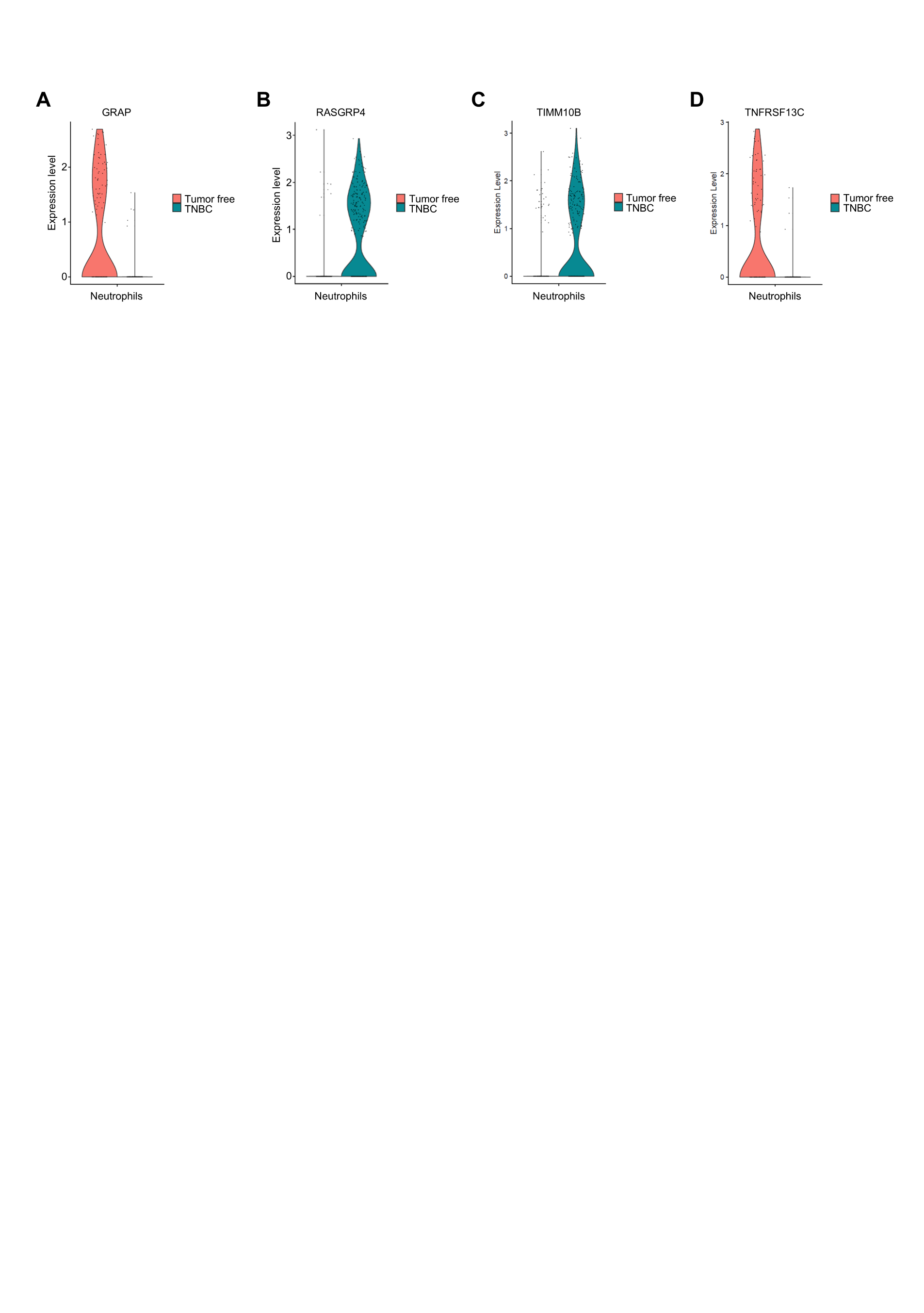


Expression patterns of the four selected genes in tumor-free and TNBC-bearing mice.

**Fig. S5**


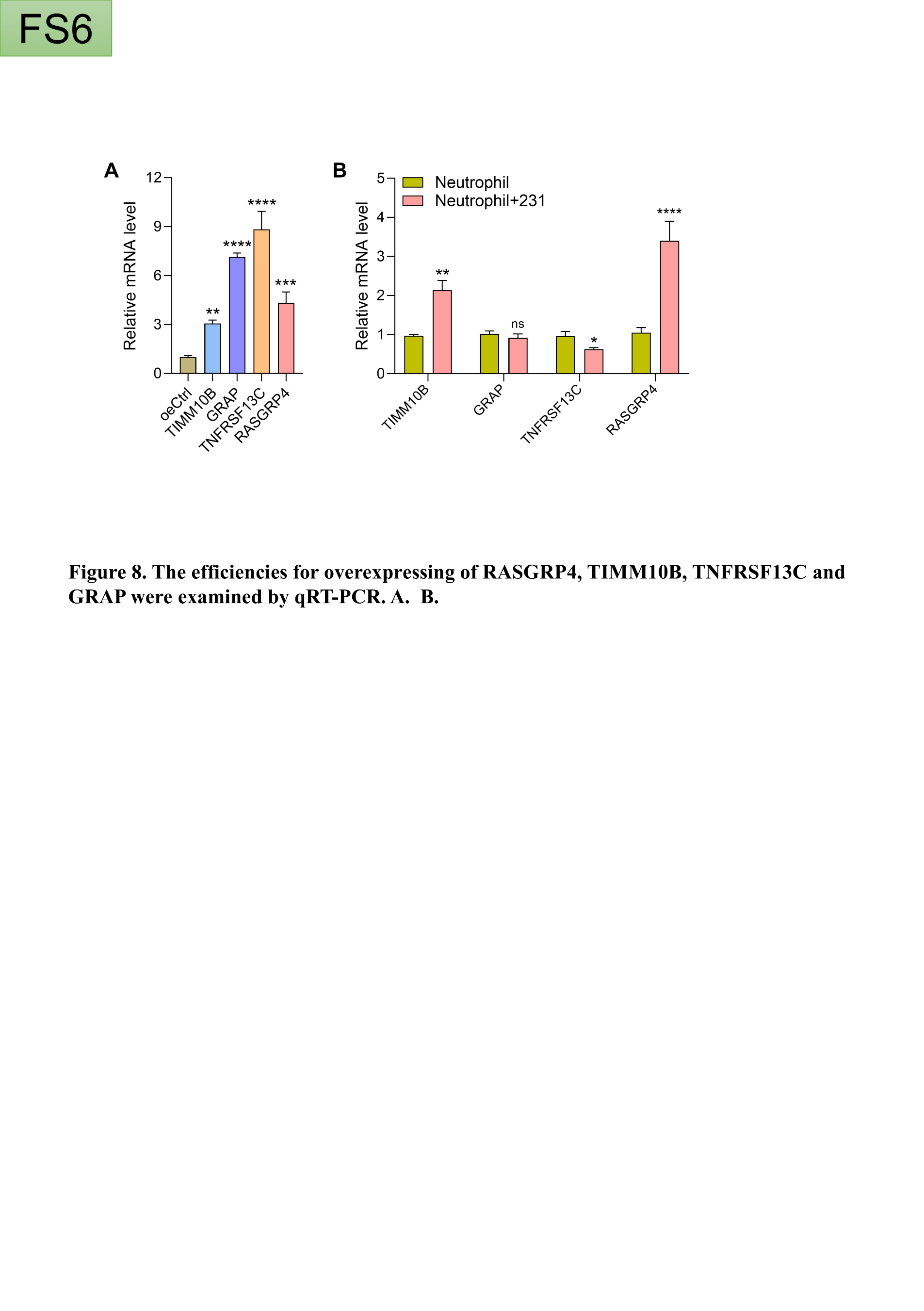


The efficiency of RASGRP4, TIMM10B, TNFRSF13C and GRAP expression was examined by qRT-PCR. (**A**). Quantified mRNA levels of TIMM10B, GRAP, TNFRSF13C and RASGRP4 after overexpressing these targeted genes in dHL-60 cells. (**B**). Quantified mRNA levels of TIMM10B, GRAP, TNFRSF13C and RASGRP4 in dHL-60 cells after coculture with MDA-MB-231 cells for 3 days.
